# Alginate Oligosaccharide Alleviates Lipopolysaccharide-Induced Apoptosis and Inflammatory Response of Rumen Epithelial Cells through NF-κB Signaling Pathway

**DOI:** 10.3390/ani14091298

**Published:** 2024-04-25

**Authors:** Xiaoyuan Qiu, Fuquan Yin, Chunmei Du, Jian Ma, Shangquan Gan

**Affiliations:** College of Coastal Agriculture Science, Guangdong Ocean University, Zhanjiang 524088, China; xxxxxhins@163.com (X.Q.); yinfuquan01@163.com (F.Y.); duchunmeim@163.com (C.D.); crazyma0411@163.com (J.M.)

**Keywords:** rumen epithelial cells, alginate oligosaccharide, inflammation, apoptosis

## Abstract

**Simple Summary:**

Alginate oligosaccharides (AOSs) have the functions of promoting cell proliferation, anti-inflammation and anti-apoptosis. In this experiment, AOS had no negative effect on cell growth. Pretreatment of AOS could improve the viability of rumen epithelial cells and help cells resist excessive apoptosis and inflammatory response caused by lipopolysaccharide (LPS). These results suggest that AOS has the potential to be used as a feed supplement to help sheep fight against apoptosis and inflammation challenges.

**Abstract:**

AOS alleviates inflammatory responses; however, whether it exerts an effect on the rumen or regulates rumen inflammatory reaction remains unknown. In this study, firstly, the ovine ruminal epithelial cells (ORECs) were treated with 0, 200, 400, 600, and 800 µg/mL AOS, hoping to explore whether AOS hurt cell health. The results showed that compared with the AOS-0 group, the AOS-400 group could significantly increase (*p* < 0.05) cell viability, reduce (*p* < 0.05) reactive oxygen species (ROS) and interleukin (IL)-6 content, and have no adverse effect on cells. Secondly, we used LPS to construct an in vitro inflammatory model of rumen epithelial cells and then explored the protective role of AOS on rumen epithelial cells. The study was divided into three groups: the control group (CON), LPS, and LPS + AOS. The results demonstrated that the LPS + AOS group significantly increased the cell viability and reduced the ROS level in comparison with the LPS group (*p* < 0.05). Pretreatment with AOS also repressed (*p* < 0.05) the secretion of IL-1β, IL-6, IL-8, and immunoglobulin (Ig)A from ORECs in the culture medium following LPS. In terms of tight junction (TJ) proteins, AOS treatment also significantly increased (*p* < 0.05) the zonula occludens 1 (ZO-1) and Occludin expression. The apoptosis rate, Caspase3, Caspase9, BAD, and BCL-2/BAX were decreased (*p* < 0.05) after AOS treatment, and the expression of BCL-2 was increased (*p* < 0.05). In addition, the expressions of Toll-like receptor 4 (TLR4), myeloid differentiation factor 88 (MyD88), and nuclear factor-κB (NF-κB) were inhibited (*p* < 0.05) with the addition of AOS. At the protein level, pretreatment of AOS decreased (*p* < 0.05) the expression of MyD88 and the phosphorylation level of inhibitor κB α (IκBα) after the LPS challenge. Taken together, our results indicated that AOS could alleviate the LPS-induced apoptosis and inflammatory response of rumen epithelial cells through the NF-κB signaling pathway, which may be a promising strategy for treating apoptosis and inflammation in sheep breeding.

## 1. Introduction

The rumen plays multifaceted and pivotal roles in the physiological processes of ruminants. It is a complex stomach compartment consisting of multiple chambers, including the reticulum, rumen, omasum, and abomasum. The rumen system in ruminants facilitates the digestion of fibrous feed and provides a diverse microbial population to promote the digestion and fermentation of feed. This process not only enhances nutrient acquisition but also produces volatile fatty acids, serving as a source of energy for the ruminants. Moreover, the rumen contributes to maintaining the balance of microbial communities, promoting nutrient absorption. Additionally, the rumen is essential for maintaining acid–base balance. For example, the short-chain volatile fatty acids produced by the rumen contribute to regulating the pH of the rumen fluid, maintaining it within an appropriate range and creating an environment most favorable for microorganisms and digestive enzymes. The rumen epithelium is critical to maintaining the normal physiological function of the rumen. However, when the rumen epithelial cells are damaged, there is a high likelihood of LPS crossing the rumen wall, entering the bloodstream, and affecting other organs such as the liver, potentially causing diseases and in severe cases, it can cause sudden death syndrome, causing animal death and economic losses [1].

Alginate oligosaccharide (AOS), mainly composed of β-D-mannuronic acid (M) and α-L-guluronic acid (G), is acidic oligosaccharides. AOS is typically obtained through the hydrolysis of alginate and exhibits superior water solubility as well as biological activity in comparison with alginate [2]. Notably, a study investigating the effect of AOS on aortic aneurysms demonstrated that AOS could modulate the Toll-like receptor 4 (TLR4)-mediated nuclear factor-κB (NF-κB) signaling pathway, effectively inhibiting the overexpression of inflammatory factors [3]. Furthermore, studies have revealed that AOS has the potential to enhance the intestinal barrier and regulate the colonic inflammatory response in mice by down-regulating the expression of interleukin (IL)-6 and IL-1β [4]. TUSI et al. [5] used PC12 cells as a model to explore the effect of AOS on H_2_O_2_-induced apoptosis. It was found that AOS could promote the expression of BCL-2, reduce the expression of BAX and the activation of Caspase3, inhibit the phosphorylation of key molecules of apoptosis, inhibit the mitochondrial-mediated caspase-dependent apoptosis response, and inhibit H_2_O_2_-induced PC12 cell death. In vitro experiments have also reported that AOS can stimulate cell proliferation [6]. In addition, WAN et al. [7] reported that AOS can alleviate the intestinal barrier damage induced by inhibiting the activation of the NF-κB signaling pathway in weaned piglets. However, the effect of AOS on the rumen and the underlying mechanisms remains unclear. Therefore, the purpose of this study was to investigate the effect of AOS on the ovine ruminal epithelial cells (ORECs) of Hu sheep, assess the effect of AOS on the LPS-induced excessive apoptosis and inflammatory response of rumen epithelial cells, and explore the potential mitigation mechanisms.

## 2. Material and Methods

### 2.1. Cell Culture

ORECs are provided by iCell Bioscience Inc. (Shanghai, China) and were identified by immunohistochemistry. Cells were cultured in DMEM/F12 containing 10% fetal bovine serum (ZETA LIFE, Menlo Park, CA, USA), 100 U/mL penicillin, and 100 μg/mL streptomycin (Gibco, Shanghai, China) at 37 °C in 5% carbon dioxide.

### 2.2. Source of AOS and LPS, Configuration of AOS

The AOS used in the study was provided by QingDao BZ Oligo Biotech Co., Ltd. (Shandong, China), with a purity of 91.8%. Firstly, 1 g of AOS powder was mixed with 10 mL DMEM to prepare an AOS stock solution with a final concentration of 100 mg/mL. Then 1 mL primary stock solution was mixed evenly with 9 mL DMEM, which was the secondary stock solution. According to the experimental needs, the secondary stock solution was diluted to 200, 400, 600, and 800 μg/mL AOS working solution.

The LPS was purified from *Escherichia coli* (055:B5) by phenol extraction.

### 2.3. Experimental Design

#### 2.3.1. Effect of AOS on the Activity and Cytokines of ORECs

According to the method of Zhao et al. [8], cells were treated with different concentrations (0, 200, 400, 600, and 800 μg/mL) of AOS and different times (6, 12, 24, and 48 h) to detect cell viability and screen AOS treatment time. Through the determination of ROS, cytokines and immunoglobulin the suitable concentration and treatment time of AOS-treated cells were selected for subsequent experiments.

#### 2.3.2. Construction of In Vitro Injury Model

The cells were exposed to different concentrations (0, 1, 10, 50, and 100 μg/mL) of LPS, and the cell viability, ROS and cytokine secretion were detected after 24 h. The time of LPS stimulating cells was based on the study of Nicole et al. [9], and then the appropriate LPS concentration was selected according to the test results of each index to construct the injury model.

#### 2.3.3. Effect of AOS on Apoptosis and Inflammatory Response

According to the experimental results of Section 2.3.1 and Section 2.3.2, the cells were randomly divided into three groups (CON group, LPS group, and AOS + LPS group). We examined the cell viability, apoptosis and inflammation-related indicators, and key pathway-related indicators of the three groups.

### 2.4. Cell Viability

In this study, the cell viability was detected by CCk-8 (ZETA LIFE, Menlo Park, CA, USA). After the cells were treated with different concentrations of additives and different times, 10 μL of CCK solution was added. After incubation for 1 h, the OD value was detected by enzyme labeling. The calculation formula is as follows.

Cell viability (%) = [A(treatment) − A(blank)]/[A(0 treatment) − A(blank)] × 100.

### 2.5. Immunoglobulin and Cytokine

The content of immunoglobulin and cytokines in each group was determined by ELISA (Jiangsu Meimian Industrial Co., Ltd., Yancheng, China).

### 2.6. ROS Detection

ELISA kit was used to detect ROS secretion when exploring the effect of AOS on cells and the establishment of an in vitro model. DCFH-DA (Beyotime Biotech Inc., Shanghai, China) probe was used to detect ROS when exploring the effect of AOS on LPS-induced ORECs injury.

### 2.7. Apoptosis Rate

The apoptosis rate was detected by Annexin V-FITC/PI (Beyotime Biotech Inc., Shanghai, China) method, and the steps were carried out according to the instructions.

### 2.8. RNA Extraction, Reverse Transcription

The cells were inoculated in a six-well plate, and the corresponding time concentrations were applied, respectively. The RNA of each group of cells was extracted by Trizol method, and then reverse transcribed into cDNA by HiScript III RT SuperMix (Vazyme, Nanjing, China).

### 2.9. RT-qPCR

The reaction was performed using the TB Green chimeric fluorescence method (Takara, China). The primers are shown in Table 1. Finally, the results were calculated by 2^−∆∆CT^.

### 2.10. Western Blot

The cells were seeded in six-well plates, and the cells were collected after the corresponding time and concentration. The protein concentration of the sample was detected by BCA method and the sample volume was calculated. The protein was fully denatured by heating at 98 °C for 8 min. SDS-PAGE protein separation gel was prepared according to the size of the target protein, and electrophoresis was performed after sample addition. After the membrane was transferred according to the wet transfer method, the rabbit primary antibodies of myeloid differentiation factor 88 (MyD88), inhibitor κB α (IκBα), p65, p-IκBα, p-p65 (1:1000) and β-actin mouse primary antibody (1:2000) were incubated at 4 °C overnight, and then horseradish peroxidase-labeled goat anti-rabbit immunoglobulin G-coupled secondary antibodies (1:4000) and goat anti-mouse immunoglobulin G-coupled secondary antibody (1:4000) at room temperature for 2 h. Finally, the strip was detected by Pierce emitter coupling logic (ECL).

### 2.11. Statistical Analysis

All results were analyzed by SPSS 26.0. One-way ANOVA and Duncan’s multi-range test were used for analysis. The results were expressed as mean ± standard error of the mean (SEM), and *p* < 0.05 indicated that the difference was significant.

## 3. Results

### 3.1. Effect of AOS on the Viability of ORECs

**Figure 1:**
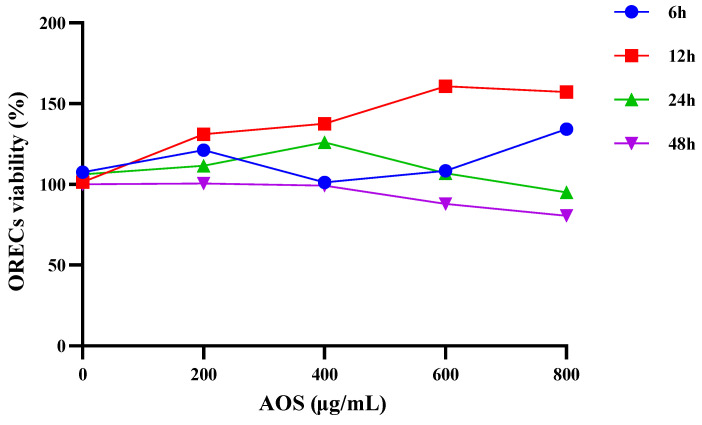
Effect of AOS on the viability of ORECs.

The cells in each group were added with the corresponding AOS concentration and cultured respectively, and the cell viability was detected by the CCK-8 method. The results are shown in Table 2 and Figure 1. It could be found that there was no significant difference in cell viability between the groups after 6 h of cell culture (*p* > 0.05). After 12 h of culture, compared with the AOS-0 group, the cell viability of each group was significantly increased (*p* < 0.05), and the cell viability of AOS-600 and AOS-800 groups was significantly higher than that of other groups (*p* < 0.05). After 24 h of culture, the cell viability of the AOS-800 group was significantly lower than that of the AOS-400 group (*p* < 0.05). After 48 h of culture, the cell viability of the AOS-600 and AOS-800 groups was significantly lower than that of other groups (*p* < 0.05). At this time, the cell viability of each group did not exceed 100%. It can be seen that the viability of ORECs is dependent on AOS concentration and culture time. The cell viability of AOS-400, AOS-600, and AOS-800 groups increased from 6 h to 48 h and then decreased. The cell viability of the AOS-600 and AOS-800 groups reached the highest at 12 h. According to the results of the CCK-8 experiment, 12 h was selected for subsequent experiments.

### 3.2. Effect of AOS on the Content of ROS, Cytokine and Immunoglobulin in ORECs

**Figure 2:**
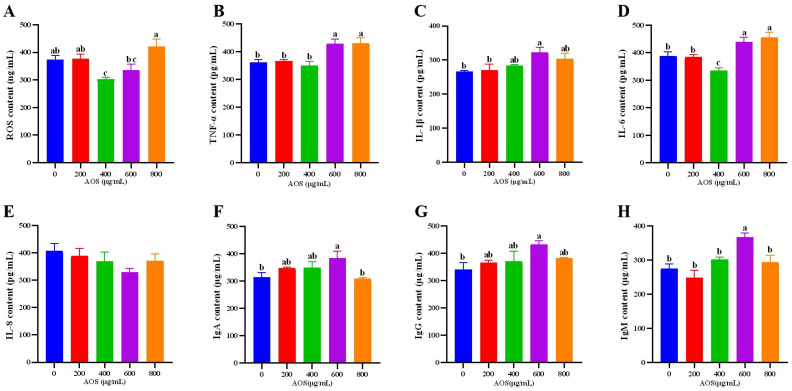
Effect of AOS on the content of ROS, cytokine and immunoglobulin in ORECs. (**A**) ROS content; (**B**) TNF-α content; (**C**) IL-1β content; (**D**) IL-6 content; (**E**) IL-8 content; (**F**) IgA content; (**G**) IgG content; (**H**) IgM content. a–c means significantly different (*p* < 0.05), no letter indicates that the difference is not significant (*p* > 0.05).

According to the results of CCK-8, the cells were cultured for 12 h after adding AOS, and the contents of cytokines and ROS in each group were detected. The results are shown in Figure 2. Compared with the AOS-0 group, the ROS content in the AOS-400 group was significantly reduced and significantly lower than that in the AOS-200 and AOS-800 groups (*p* < 0.05). The content of ROS in the AOS-600 group was significantly lower than that in the AOS-800 group (*p* < 0.05) (Figure 2E).

After the cells were treated with AOS-600 and AOS-800 groups for 12 h, the tumor necrosis factor-α (TNF-α) content of the two groups was significantly higher than that of AOS-0, AOS-200, and AOS-400 groups (*p* < 0.05) (Figure 2A). As shown in Figure 2B, compared with the AOS-0 group, the IL-1β content in the AOS-600 group was significantly increased (*p* < 0.05). As shown in Figure 2C, compared with the AOS-0 group, the IL-6 content in the AOS-400 group was significantly decreased (*p* < 0.05), while the IL-6 content in AOS-600 and AOS-800 groups was significantly increased (*p* < 0.05). The IgA content of AOS-600 was significantly higher (*p* < 0.05) than that of AOS-0 and AOS-800. In terms of IgM, the content of AOS-600 group was significantly higher than that of other groups (*p* < 0.05). The content of IgG in AOS-600 group was significantly higher (*p* < 0.05) than that in AOS-0 group.

Therefore, this study decided to select the AOS addition amount of 400 μg/mL for subsequent experiments.

### 3.3. Establishment of ORECs Inflammatory Injury Model

**Figure 3:**
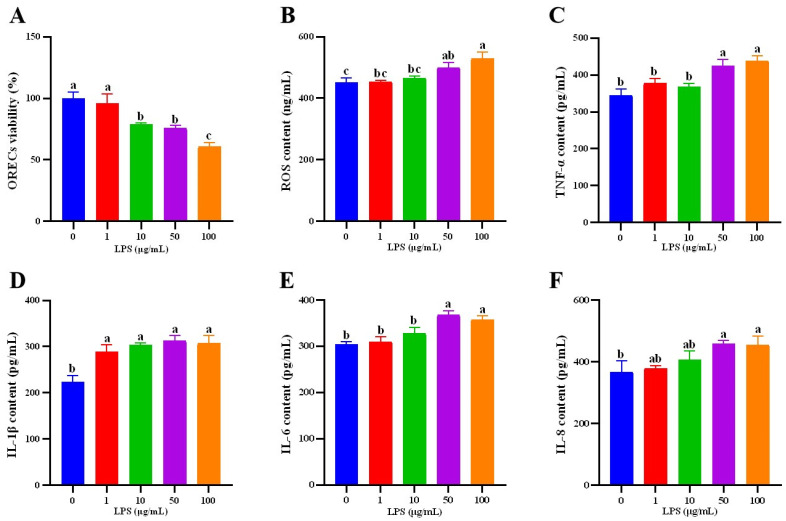
Effect of LPS on the viability, ROS, immunoglobulin, and cytokine content of ORECs. (**A**) ORECs viability; (**B**) ROS content; (**C**) TNF-α content; (**D**) IL-1β content; (**E**) IL-6 content; (**F**) IL-8 content. a–c means significantly different (*p* < 0.05).

To establish an in vitro inflammatory injury model, this study then studied the effects of different concentrations (0, 1, 10, 50, and 100 µg/mL) of LPS on cells for 24 h. The cells in each group were cultured for 24 h after LPS was added, and the cell viability was detected. The results are shown in Figure 3A. The cell viability of the LPS-10, LPS-50, and LPS-100 groups was significantly lower than that of the LPS-0 and LPS-1 groups, and the cell viability of the LPS-100 group was significantly lower than that of LPS-10 and LPS-50 groups (*p* < 0.05), There was no significant difference between the other groups (*p* > 0.05).

This experiment then examined the changes in ROS. Figure 3B is the result of ROS content detection. Compared with the LPS-0 group, the ROS content of the LPS-50 and LPS-100 groups was significantly increased (*p* < 0.05), and the ROS content of the LPS-100 group was significantly higher than that of other addition groups (*p* < 0.05).

Cytokines such as IL-6 are important indicators for the success of inflammatory model construction. As shown in Figure 3C,E, the contents of TNF-α and IL-6 in the LPS-50 and LPS-100 groups were significantly higher than those in the other groups (*p* < 0.05). As shown in Figure 3D, the IL-1β content of the LPS-0 group was significantly lower than that of the other groups (*p* < 0.05). As shown in Figure 3F, the IL-8 content in the LPS-50 and LPS-100 groups was significantly higher than that in the LPS-0 group (*p* < 0.05).

Based on the above results, this study selected the LPS concentration of 50 μg/mL for 24 h to construct an in vitro inflammatory injury model.

### 3.4. Effects of AOS on LPS-Induced ORECs Viability, Immunoglobulins, Cytokines and Tight Junction Proteins

According to the above experimental results, the additional amount of AOS was 400 μg/mL and the action time was 12 h. The additional amount of LPS was 50 μg/mL. The experiment was divided into (1) control group (CON): normal medium was cultured for 12 + 24 h; (2) LPS group: cells were cultured in normal medium for 12 h, and then treated with 50 μg/mL LPS for 24 h. (3) AOS + LPS group: cells were pre-cultured with AOS final concentration of 400 μg/mL medium for 12 h, and then treated with medium containing 50 μg/mL LPS for 24 h.

**Figure 4:**
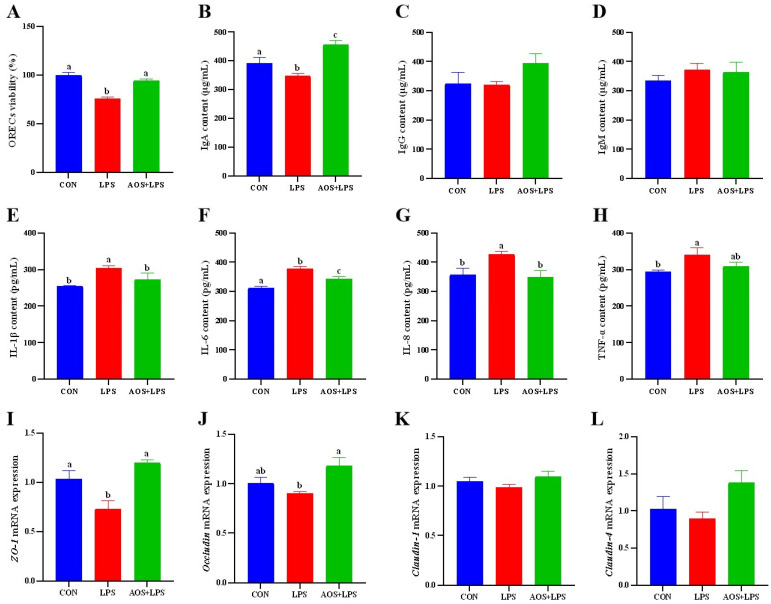
Effect of AOS on LPS-induced inflammation in ORECs. (**A**) ORECs viability; (**B**) IgA content. (**C**) IgG content; (**D**) IgM content; (**E**) IL-1β content; (**F**) IL-6 content; (**G**) IL-8 content; (**H**) TNF-α content; (**I**) *ZO-1* mRNA expression; (**J**) *Occludin* mRNA expression; (**K**) *Cludin-1* mRNA expression; (**L**) *Cludin-4* mRNA expression. a–c means significantly different (*p* < 0.05), no letter indicates that the difference is not significant (*p* > 0.05).

The cells were cultured according to the time and concentration, and the cell viability of each group was detected. The results are shown in Figure 4A. Compared with the CON group, the cell viability of the LPS group was significantly decreased (*p* < 0.05). There was no significant difference in cell viability between the AOS + LPS group and the CON group (*p* > 0.05).

Figure 4B–D is the immunoglobulin test results. As shown in Figure 4B, there was no significant difference in immunoglobulin (Ig)A content between the CON group and the AOS + LPS group (*p* > 0.05), but both were significantly lower than the LPS group (*p* < 0.05) (Figure 4D,E). As shown in Figure 4H, the content of TNF-α in the LPS group was significantly higher than that in the CON group (*p* < 0.05). As shown in Figure 4E,G, the levels of IL-1β and IL-8 in the CON group and AOS + LPS group were not significantly different (*p* > 0.05), but were significantly lower than those in the LPS group (*p* < 0.05). As shown in Figure 4F, compared with the CON group, the IL-6 content in the LPS group and the AOS + LPS group was significantly increased (*p* < 0.05), and the LPS group was significantly higher than the AOS + LPS group (*p* < 0.05).

As shown in Figure 4I, there was no significant difference in the gene expression of *zonula occludens 1* (*ZO-1*) between the CON group and the AOS + LPS group (*p* > 0.05), but both were significantly higher than the LPS group (*p* < 0.05). The gene expression of *Occludin* in the AOS + LPS group was significantly higher than that in the LPS group (*p* < 0.05) (Figure 4J).

### 3.5. Effect of AOS on LPS-Induced ORECs ROS Content 

**Figure 5:**
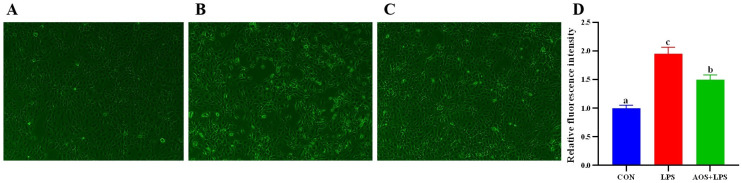
Effect of AOS on LPS-induced ORECs ROS contect. (**A**–**C**) Fluorescence images of ROS in CON, LPS, and AOS + LPS groups. (**D**) relative fluorescence intensity. a–c means significantly different (*p* < 0.05).

Figure 5D shows the relative fluorescence intensity of ROS in each group. Compared with the CON group, the relative fluorescence intensity of AOS + LPS group was significantly increased (*p* < 0.05), and the relative fluorescence intensity of LPS group was significantly increased (*p* < 0.05) and significantly higher than that of AOS + LPS group (*p* < 0.05).

### 3.6. Effects of AOS on LPS-Induced ORECs Apoptosis Rate and Apoptosis-Related Genes

**Figure 6:**
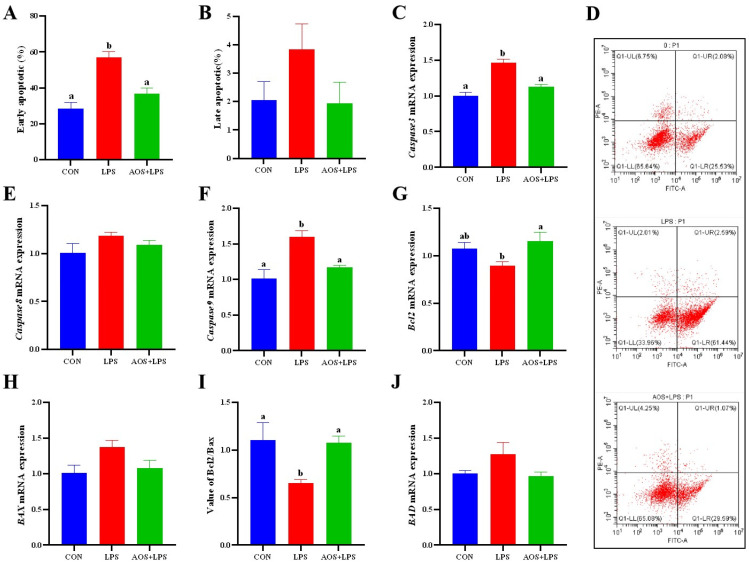
Effects of AOS on LPS-induced ORECs apoptosis rate and apoptosis-related genes expression. (**A**) Early apoptosis; (**B**) Late apoptosis; (**D**) results of cell apoptosis; (**C**,**E**–**J**) Expression of apoptosis-related genes. a, b means significantly different (*p* < 0.05), no letter indicates that the difference is not significant (*p* > 0.05).

As shown in Figure 6A, the early apoptosis rate of the LPS group was significantly higher than that of the CON group and AOS + LPS group (*p* < 0.05). As shown in Figure 6C,F,J, compared with the CON group, the expression of *Caspase3*, *Caspase9* and *BAD* genes in the LPS group increased significantly (*p* < 0.05). The expression of the *BCL-2* gene in the LPS group was significantly lower than that in the AOS + LPS group (*p* < 0.05), and there was no significant difference between the two groups and the CON group (*p* > 0.05) (Figure 6G). The ratio of Bcl2/Bax in the CON group and AOS + LPS group was significantly higher than that in the LPS group (*p* < 0.05) (Figure 6I).

### 3.7. Effects of AOS on LPS-Induced ORECs NF-κB Signaling Pathways

**Figure 7:**
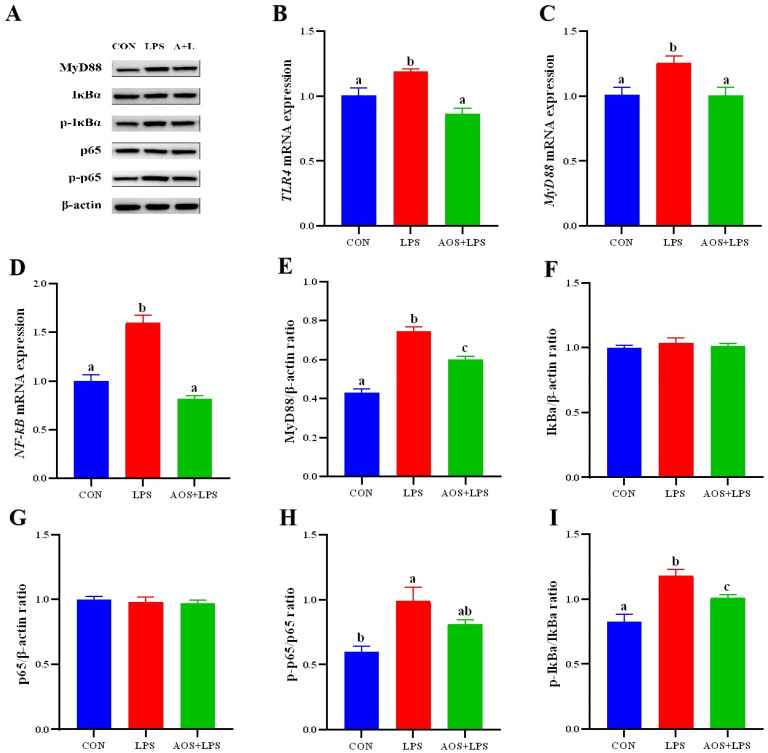
Effect of AOS on NF-κB pathway-related genes and proteins in LPS-induced ORECs. (**A**) Western blot results; (**B**–**D**). *TLR4*, *MyD88*, and *NF-κB* mRNA expression; (**E**–**G**) Relative expression of MyD88, IκBα and p65 protein; (**H**) Phosphorylation level of p65; (**I**) Phosphorylation level of IκBα. a–c means significantly different (*p* < 0.05), no letter indicates that the difference is not significant (*p* > 0.05).

In order to further explore the protective mechanism of AOS on cells, the mRNA expression of *TLR4*, *MyD88*, and *NF-κB* was detected in this experiment. According to Figure 7B, the expression of the *TLR4* gene in the AOS + LPS group was significantly lower than that in the LPS group (*p* < 0.05). Figure 7C,D reflect the gene expression of *MyD88* and *NF-κB*. There was no significant difference between the CON group and the AOS + LPS group (*p* > 0.05) and both were significantly lower than the LPS group (*p* < 0.05). According to the results of gene expression detection, it can be found that the expression of key genes in the *NF-κB* pathway changes with the treatment of AOS and LPS. To confirm that AOS can regulate the inflammatory response caused by LPS by inhibiting the NF-κB pathway, the expression of key proteins in this pathway was finally detected. The results are shown in Figure 7 LPS group significantly increased the protein expression of MyD88 and the phosphorylation level of IκBα (*p* < 0.05) (Figure 7E,I). The protein expression of MyD88 and the phosphorylation level of IκBα in the AOS + LPS group were significantly lower than those in the LPS group (*p* < 0.05). The protein expression of MyD88 and the phosphorylation level of IκBα in both groups were higher than those in the CON group (*p* < 0.05). The protein expression of MyD88 and the phosphorylation level of IκBα in both groups were higher than those in the CON group (*p* < 0.05). The phosphorylation level of p65 in the LPS group was significantly increased (*p* < 0.05) (Figure 7H).

## 4. Discussion

As a low-molecular-weight oligosaccharide, AOS has a variety of biological functions. However, the current research on AOS mainly focuses on poultry, piglets, and aquatic animals [7,10,11]. The effect of AOS on ruminants was only reported in Angus beef cattle, which showed that AOS promoted the healthy growth of beef cattle by improving the structure of rumen microbial flora [12]. At present, it is not clear whether AOS has an effect on sheep, so we first explored the effect of AOS on rumen epithelial cells. CCK-8 results showed that the effect of AOS on cell viability depended on culture time and concentration. Li et al. also found that the viability of bovine mammary epithelial cells demonstrates a concentration-dependent pattern in response to treatment with different concentrations of curcumin [13]. In our experiment, when the culture time was 12 h, the cell viability of each group was higher than that of the AOS-0. Therefore, this study further explored the effect of AOS on ORECs at 12 h. ROS is a metabolic product of the body, which is a group of unstable molecules, including hydrogen peroxide, hydroxide, singlet oxygen, and superoxide [14]. Excessive ROS can damage intracellular proteins, lipids, and nucleic acids, resulting in oxidative damage to the body and inducing apoptosis. Moreover, the ROS could destroy mitochondria, induce more ROS release, and further aggravate the production of inflammatory cytokines [15,16,17]. Our results show that the AOS-400 group can reduce the content of ROS in cells, which suggests that AOS at this concentration can alleviate oxidative damage, reduce apoptosis, or reduce the content of inflammatory cytokines. Therefore, we further detected the cytokines. The results show that the AOS-400 group can reduce the secretion of IL-6, which suggests that AOS has the potential to alleviate inflammatory reactions. However, when the concentration of AOS reaches or exceeds 600 μg/mL, the ability of AOS to alleviate inflammation will disappear, and even adversely affect cells. Based on current research findings, it is hypothesized that this association may be related to mannuronic acid; however, further investigations are required to elucidate this relationship [18]. In conclusion, when 400 μg/mL AOS was added for 12 h, it was beneficial to the growth of ORECs and against inflammatory challenges, so we chose this time and dose for subsequent experiments.

LPS serves as the endotoxin found in Gram-negative bacteria, which can bind to specific pattern recognition receptors and activate the NF-κB signaling pathway, accompanying increasing the content of inflammation-related cytokines such as IL-1β, IL-6, IL-8, and TNF-α. For example, the content of IL-1β, IL-6, and TNF-α in bovine rumen epithelial cells (BRECs) increased under LPS stimulation [19]. Therefore, LPS is often used to induce inflammatory models in vitro and in vivo [20]. In this study, LPS was chosen to establish the inflammatory model of ORECs. After research, we found that when the LPS additive amount was 50 μg/mL and 100 μg/mL, the activity of ORECs was decreased, and the contents of ROS, TNF-α, and IL-6 were increased. The changes in these indexes indicate that the cells have an inflammatory reaction under the stimulation of LPS. Moreover, based on the results of cell viability (cell viability was 76% when 50 μg/mL LPS stimulated cells, and 61% when 100 μg/mL LPS stimulated cells), we decided to select the 50 μg/mL LPS to induce an inflammatory model. In general, this study constructed an in vitro inflammatory model with an LPS addition of 50 μg/mL and a stimulation time of 24 h.

Based on the above results, this study then explored the ability of AOS to alleviate the inflammatory response of ORECs induced by LPS. AOS has been reported to have the ability to stimulate cell migration and proliferation, which may be related to the guluronic acid at the end of AOS. Peripheral guluronic acid has more affinity for receptors on endothelial cells [6,21,22]. The results of this study also once again demonstrated the ability of AOS to stimulate cell proliferation. The results showed that AOS could increase cell viability and reduce ROS content in the LPS-induced rumen epithelial cell inflammation model.

The rumen epithelial tight junction (TJ) is distributed at the top of intercellular space, and plays a key role in maintaining the polarity of epithelial cells, regulating the permeability of the epithelial barrier, and preventing toxin translocation [23,24,25]. TJ is mainly composed of Claudin and Occludin. These proteins mediate adhesion function and are linked to ZO-1, which can repair tight junctions [26,27]. Occludin is now considered to be the key to maintaining TJ macromolecular channels. Studies have shown that the reduction in Occludin leads to a selective or preferential increase in macromolecular flux on intestinal epithelial cells, both in vivo and in vitro [28]. It is reported that LPS can lead to changes in the expression and distribution of TJ proteins, reduce the transport capacity of sodium ion paracellular pathways, and ultimately cause epithelial swelling and barrier function damage [29]. The damaged TJ barrier is an important pathogenic factor of gastrointestinal inflammatory diseases. Excessive cytokines such as TNF-α and IL-1β are one of the reasons for the increased permeability of TJ. The increase in TJ permeability induced by them is mediated by the activation of NF-κB and myosin light chain kinase (MLCK) gene [30,31]. Therefore, we focused more closely on the changes of TJ proteins. The results of this study showed that compared with the LPS group, AOS increased the gene expression of *ZO-1* and *Occludin*. Wan et al. also reported that AOS could increase the expression of Occludin in porcine intestinal barrier damage caused by enterotoxigenic *Escherichia coli* (ETEC), thereby protecting porcine intestinal epithelial cells from damage [7]. These findings implied that AOS could enhance the rumen physical barrier by increasing the expression of *ZO-1* and *Occludin*, thereby protecting rumen epithelial cells from damage. This also suggests that AOS is likely to reduce the secretion of pro-inflammatory factors by mediating the NF-κB cell pathway, participating in the regulation of inflammatory response, and protecting the TJ barrier. The detection results of pro-inflammatory cytokines and NF-κB signaling pathways also confirmed this statement.

Usually, cytokines provide valuable information for the assessment of cell health. TNF-α is widely expressed and is a key mediator and regulator of the immune response. It can activate the NF-κB signaling pathway, ultimately reduce the expression of ZO-1, destroy the protein network of intestinal epithelial cells, and aggravate the inflammatory response [32,33]. As pro-inflammatory cytokines, IL-6 and IL-1β are key mediators of the inflammatory response, which exacerbates the damage of chronic diseases and acute tissue damage. Among them, IL-6 can be induced by TNF-α or LPS, resulting in acute inflammatory response, and it can also increase the secretion of IgA in mucosal response [34,35,36]. These cytokines (TNF-α, IL-6, and IL-1β) may be induced by IL-8. However, including IL-8, they are regulated by the NF-κB signaling pathway [37]. Then we detected the content of these classical cytokines. The results showed that AOS reduced the levels of IgA, IL-6, IL-1β, and IL-8. Zhang et al. also reported that AOS could reduce the levels of TNF-α, IL-1, and IL-6 in mouse macrophages stimulated by LPS [38]. All these indicate that AOS can alleviate the inflammatory response caused by LPS.

Inflammatory response and apoptosis are complex interactions. When an inflammatory reaction occurs, inflammatory cells are released, and they trigger apoptosis and clear damaged cells through a series of reactions. However, when apoptosis is abnormal, the accumulation of damaged cells cannot be cleared in time, and excessive stimulation of cells to secrete inflammatory factors leads to increased inflammatory response and further aggravates the occurrence of apoptosis. ROS is an important regulator of oxidative signal transduction and cell homeostasis. When NF-κB and MAPK are activated, a small amount of ROS is increased, which can be used as a signal transduction for normal physiological activities of cells [39]. However, excessive ROS can cause DNA damage and apoptosis. This study found that LPS caused an increase in ROS levels, and AOS alleviated this phenomenon. Corresponding to the early apoptosis rate of cells, LPS significantly increased the apoptosis rate of ORECs, but under the intervention of AOS, the early apoptosis of cells was alleviated. Zhao et al. [8] reported that AOS could reduce the expression levels of *Caspase3* and *BAX*, increase the expression level of *BCL-2*, and protect human umbilical vein endothelial cells (HUVECs) from apoptosis induced by H_2_O_2_. The results of this study showed that AOS could increase the expression level of *BCL-2*, increase the value of BCL-2/Bax, and protect ORECs from excessive apoptosis induced by LPS by reducing the expression levels of *Caspase3* and *Caspase9* and the expression level of pro-apoptotic factor *BAD*.

It is well known that LPS can activate the NF-κB signaling pathway mediated by TLR4, which is mainly involved in the regulation of inflammatory response. Generally, inhibitor κB (IκB) binds to NF-κB, making NF-κB unable to play a role. NF-κB protein is usually a dimeric protein formed by p65/p50. MyD88, as an adaptor protein, receives the stimulation of cytokines such as IL-1β to ubiquitinate TAK1, IκBα phosphorylation and leads to its degradation, activates NF-κB dimer protein, and regulates the release of cytokines such as TNF-α, IL-1β, and IL-8. This is a classical pathway of NF-κB signaling pathway activation. The phosphorylation of p65 and IκBα is a marker of NF-κB pathway activation [40,41,42,43]. To investigate the functional role of AOS in inflammatory response. ORECs were exposed to LPS, in the presence or absence of AOS. In agreement with previous studies, LPS treatment led to marked activation of the NF-κB signaling pathway. Interestingly, LPS-induced activation of NF-κB was mitigated by AOS. We found that AOS pretreatment decreased the gene expression levels of *TLR4*, *MyD88*, and *NF-κB*. Western blot detection further confirmed this result. Western blot results showed that AOS treatment significantly reduced the protein expression level of MyD88 and the phosphorylation level of IκBα, and the phosphorylation level of p65 was not significantly different from that of the CON group. These results suggest that AOS alleviates LPS-induced cellular apoptosis and inflammatory response by inhibiting IκBα degradation, inhibiting NF-κB signaling pathway activation and reducing the release of inflammatory factors.

## 5. Conclusions

In summary, 400 μg/mL AOS was beneficial to the healthy growth of ORECs when cells were cultured for 12 h, and AOS can lessen the excessive apoptosis and inflammatory response through the interference of the NF-κB signaling pathway. AOS was able to maintain the immune homeostasis of the rumen, showing a potency to be used in treating excessive apoptosis and inflammation. This also suggests that adding AOS to the animal’s daily diet may help animals resist the challenge of inflammation.

## Figures and Tables

**Table 1:** Primers for real-time PCR analyses.

Gene	Primer Sequence, 5′to 3′	Accession Number	Size (bp)
*β-actin*	F:ACAGCCGAGCGGGAAAT	XM_004013078.5	215
	R:ATGCCACAGGACTCCATACC		
*TLR4*	F:AAAGAACTTGGAGGAGGGC	XM_042242671.1	141
	R:TGCTGGGACACCACGAC		
*MyD88*	F:CCCATCAAGTACAAGCCAATG	NM_001166183.1	109
	R:CGAGGCGAGTCCAGAACC		
*NF-kB*	F:GTTTACGCCTGATGATTTGC	XM_042251202.1	180
	R:TTGGAGGGAGCGGGACT		
*ZO-1*	F:TGAACGCAAGTTTGAAAGTCC	XM_042235171.1	288
	R:CGGGCAGTAGCCACCAC		
*Occludin*	F:CGGAGGAAGTGCCTTTGG	XM_012096797.4	101
	R:CCTTTGCCGCTCTTGGAT		
*Claudin-1*	F:GGGGCTGTGGATGTCGT	NM_001185016.1	202
	R:CTTCTGTGCCTCGTCGTCT		
*Claudin-4*	F:TTCATCGGCAGCAACATCG	NM_001185017.2	189
	R: CAACAGCACGCCAAACACG		
*BCL-2*	F:CGCATCGTGGCCTTCTT	XM_027960877.2	291
	R:TCCCAGCCTCCGTTGTC		
*BAX*	F:TCCGACGGCAACTTCAA	XM_027978594.2	242
	R:AGCACTCCAGCCACAAAGA		
*BAD*	F:TCCCAGAGTTTGAGCAGAGTGA	XM_042238345.2	428
	R:GCTAGGGCTTTGTCGCATTT		
*Caspase3*	F:TGGAACCAATGGACCCG	XM_027962551.2	247
	R:CTTTGAGTTTCGCCAGGAA		
*Caspase9*	F:ACCAGCAGACAAGCAGCAA	XM_042257438.1	253
	R:CAGTGAATCCTCCAGAACCAA		
*Caspase8*	F:AATGCCCTTCCCTTGTCG	XM_042244960.1	173
	R:CAGCAGAAAGTCAGCCTCAT		

**Table 2:** Effect of AOS on the viability of ORECs (%).

AOS (µg/mL)	Time (h)				*p*
	6	12	24	48	
0	107.66 ± 10.04	101.39 ± 5.02 ^c^	106.31 ± 8.52 ^ab^	100.00 ± 4.52 ^a^	0.423
200	121.29 ± 12.99 ^AB^	131.00 ± 14.48 ^Ab^	111.60 ± 18.02 ^ABab^	100.56 ± 8.24 ^Ba^	0.049
400	101.20 ± 14.20 ^B^	137.59 ± 9.18 ^Ab^	126.12 ± 23.27 ^Aa^	99.23 ± 7.85 ^Ba^	0.008
600	108.37 ± 10.67 ^B^	160.80 ± 13.52 ^Aa^	106.97 ± 6.94 ^Bab^	87.97 ± 6.76 ^Cb^	<0.001
800	134.21 ± 45.63 ^A^	157.33 ± 11.16 ^Aa^	95.01 ± 3.24 ^Bb^	80.51 ± 5.64 ^Bb^	0.002
*p*	0.313	<0.001	0.083	0.002	

*p* < 0.05 were considered to be statistically significant. Values in the same row with different letters/columns (A,B,C/a,b,c) are significantly different.

## Data Availability

The analyzed data from this study are available from the corresponding author on request.
